# Clinical Application of Multigene Panels: Challenges of Next-Generation Counseling and Cancer Risk Management

**DOI:** 10.3389/fonc.2015.00208

**Published:** 2015-09-29

**Authors:** Thomas Paul Slavin, Mariana Niell-Swiller, Ilana Solomon, Bita Nehoray, Christina Rybak, Kathleen R. Blazer, Jeffrey N. Weitzel

**Affiliations:** ^1^Division of Clinical Cancer Genetics, Department of Medical Oncology, City of Hope, Duarte, CA, USA

**Keywords:** multigene panels, hereditary breast cancer, hereditary colon cancer, genetic counseling, next-generation sequencing, hereditary cancer panel

## Abstract

**Background:**

Multigene panels can be a cost- and time-effective alternative to sequentially testing multiple genes, especially with a mixed family cancer phenotype. However, moving beyond our single-gene testing paradigm has unveiled many new challenges to the clinician. The purpose of this article is to familiarize the reader with some of the challenges, as well as potential opportunities, of expanded hereditary cancer panel testing.

**Methods:**

We include results from 348 commercial multigene panel tests ordered from January 1, 2014, through October 1, 2014, by clinicians associated with the City of Hope’s Clinical Cancer Genetics Community of Practice. We also discuss specific challenging cases that arose during this period involving abnormalities in the genes: *CDH1*, *TP53*, *PMS2*, *PALB2*, *CHEK2*, *NBN*, and *RAD51C*.

**Results:**

If historically high risk genes only were included in the panels (*BRCA1*, *BRCA2*, *MSH6*, *PMS2*, *TP53*, *APC*, *CDH1*), the results would have been positive only 6.2% of the time, instead of 17%. Results returned with variants of uncertain significance (VUS) 42% of the time.

**Conclusion:**

These figures and cases stress the importance of adequate pre-test counseling in anticipation of higher percentages of positive, VUS, unexpected, and ambiguous test results. Test result ambiguity can be limited by the use of phenotype-specific panels; if found, multiple resources (the literature, reference laboratory, colleagues, national experts, and research efforts) can be accessed to better clarify counseling and management for the patient and family. For pathogenic variants in low and moderate risk genes, empiric risk modeling based on the patient’s personal and family history of cancer may supersede gene-specific risk. Commercial laboratory and patient contributions to public databases and research efforts will be needed to better classify variants and reduce clinical ambiguity of multigene panels.

## Introduction

An interdisciplinary medical practice that employs a growing arsenal of genetic and genomic tools to identify individuals and families with inherited cancer risk (1), genetic cancer risk assessment (GCRA) enables informed choices about cancer screening (2–4), surgical (5–9), and chemopreventive risk management options (10–14), as well as genetically targeted cancer treatment therapies (15, 16). Although genetic counseling and testing driven by syndromic features, with testing focused on one or a few high penetrance cancer predisposition genes, has been the standard of care, technical advances have upended the well-established paradigms. National guidelines now include discussion of hereditary cancer panels inclusive of multiple genes as a potentially cost- and time-effective alternative to sequentially testing multiple single genes associated with a given phenotype; or when atypical family presentations, or limited family structure make it difficult to use family history alone to determine the most appropriate gene(s) to test (17, 18).

**Figure 1:**
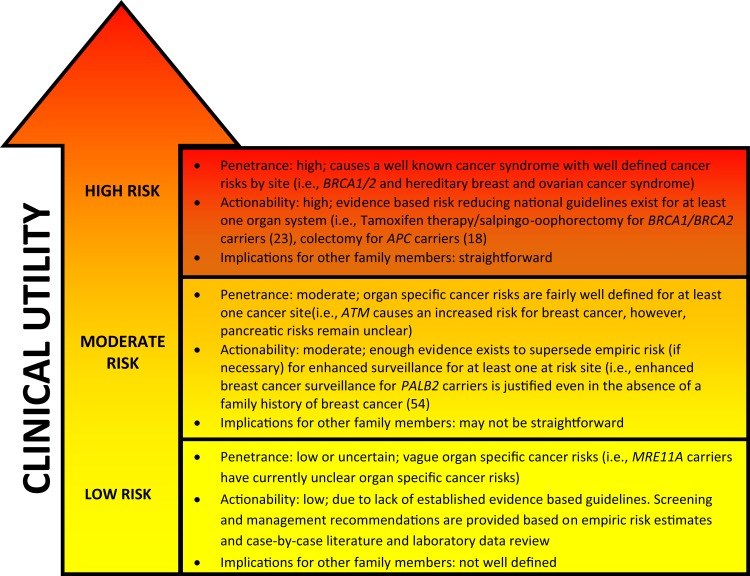
**General characteristics of genetic cancer risk groups**. Genetic risk categories are shown with an adjacent matched color descriptor noting the general features specific to each risk tier. Quantification of risk with a categorization of genes in each tier is provided in Table 1. Clinical utility (arrow) increases with higher cancer risk predisposition. The arrow gradient denotes the potential significant overlap between the tiers. Clinical utility and refined risk scores may improve in the future, especially for low and moderate risk genes (19). Penetrance, actionability, and implications for family members have been simplified for conceptual use.

Moving beyond single-gene testing has unveiled new challenges to the clinician involved in providing GCRA. Since the implementation of multigene panels, significant gaps in our gene-specific phenotypic knowledge base have been identified. The prevalence of variants of uncertain significance (VUS), unexpected findings, such as “off-phenotypic-target” gene mutations, and pathogenic findings in low and moderate risk genes challenge the established counseling repertoire. Even in the case of mutations in highly actionable genes, expanded panel testing can lead to unexpected findings. The purpose of this article is to illustrate some of the challenges and opportunities associated with expanded hereditary cancer panel tests, many of which include both well-characterized and lesser known cancer-associated genes. We also provide a conceptual framework according to evidence for clinical utility to help classify low, moderate, and high risk cancer predisposing genes (Figure 1; Table 1).

**Table 1:** **Breast, colorectal, and ovarian cancer risk estimates by monoallelic germline mutation**.

Cancer site	High risk (odds^θ^ ≥5.0)	Moderate risk (≥2.0 odds^θ^ <5.0)	Low risk (≤2.0 odds^θ^ ≥1.0 or growing evidence of association)
Breast (female)	*BRCA1* (20), *BRCA2* (20), *CDH1* (21), *PTEN* (22), *STK11* (23, 24), *TP53* (25)	*ATM* (26, 27), *BRIP1* (28), *CHEK2* (29, 30), *PALB2* (31, 32)	*BAP1* (33), *BARD1* (34, 35), *RAD50* (36, 37), *RAD51C* (38), *RAD51D* (39, 40), *MRE11A* (36), *MUTYH* (41), *NBN* (42, 43), *XRCC2* (44, 45)
Colorectal	*APC* (46), *BMPR1A* (47), ^ψ^*EPCAM* (48), *MLH1* (49), *MSH2* (49), *MSH6* (49, 50), *MUTYH (51), *PMS2* (52), *SMAD4* (47), *STK11* (53)	*CHEK2* (54, 55), *PTEN* (56), *TP53* (25)	*CDH1* (57, 58), *EXO1* (59), *GALNT12* (60, 61), *MUTYH* (62, 63), *POLD1* (64), *POLE* (64)
Ovary	*BRCA1* (65), *BRCA2* (65), *MLH1* (66), *MSH2* (66), *STK11* (24)	*MSH6* (66), *PALB2* (32, 65), *RAD51C* (65, 67), *RAD51D* (39)	*BARD1* (65, 68), *BRIP1* (65), *CHEK2* (65), *MRE11A* (65), *MUTYH* (69), *NBN* (65), *RAD50* (65), *TP53* (65)

Due to study design variation, genetic risk categorization was extrapolated from odds ratios, relative risks, cumulative, or absolute cancer risks and presented as an estimate of the generalized odds (θ) over the baseline population for organ specific cancer risk. Genes in each category are in alphabetical order. Please see individual key reference for specific risk estimate method used. When study discrepancy, or wide reported confidence intervals were reported, expert opinion was used for the final risk categorization. The list is not exhaustive for breast, colorectal, and ovarian cancer predisposition. More studies, especially on moderate and low risk category genes will be needed to better clarify the associated cancer risks and penetrance. Single nucleotide polymorphism studies, which could add hundreds of gene and locus associations to the low risk category, were not included (70). Penetrance and expressivity can widely vary with specific mutations. Asterisk (*) denotes *MUTYH* biallelic mutation. (^ψ^) denotes deletions only affecting transcription of *MSH2*.

Since the advent of lower-cost next-generation sequencing, multigene panels now include 5–60 genes (71–74). Some panels are phenotype specific and include breast or colon cancer risk genes, whereas others cover a broad spectrum of cancers and are marketed for expanded pan-cancer genetic risk assessment. The driver of cancer genetic testing has historically been clinical utility, based on sufficient evidence to support significant changes in patient and/or family screening and risk management recommendations (1, 74–78). Virtually, all multigene panels include “high penetrance genes” associated with multiple interrelated phenotypes. Some of these genes are specific to breast cancer risk, some specific to colon cancer risk, some specific to both, and/or other organ system risk (Table 1). However, as shown by our case reports below, expanded panel testing even for these genes can lead to unexpected findings. Furthermore, the addition of many moderate to low risk genes on panels can make it challenging to develop personalized management guidelines for the patient and family when a pathogenic mutation is found, since the phenotypic spectrum and penetrance are less defined, or unknown, at this time.

## Materials and Methods

### Patient selection

**Figure 2:**
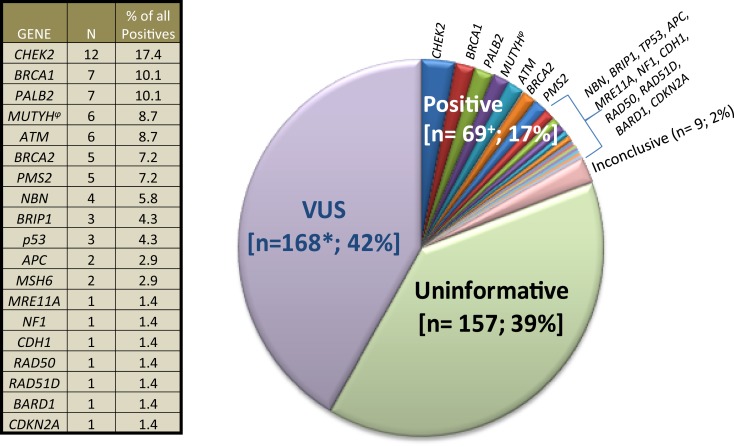
**Clinical cancer genetics community of practice experience with multigene panel tests**. 403 results of 348 commercial multigene panel tests ordered by Clinical Cancer Genetics Community of Practice clinicians between January 1, 2014, through October 1, 2014 are depicted. “VUS” means variant of uncertain significance. “Uninformative” refers to negative panel testing results. “Inconclusive” refers to the laboratories inability to classify the result into other categories at the present time. The plus symbol (+) denotes that six patients had mutations in ≥1 gene. Asterisk (*) denotes that 35 patients had ≥1 VUS. The side table shows the number of individual positive gene mutations found. (φ) denotes that five MUTYH cases were monoallelic, whereas one case was biallelic.

The City of Hope Division of Clinical Cancer Genetics (CCG) includes a cancer screening and prevention program, cancer genetics education program, and research program. The Clinical Cancer Genetics Community of Practice (CCGCoP) was established as a multifaceted program of GCRA training and ongoing distance-mediated practice support for community-based clinicians, funded by the NCI (R25CA171998) (79, 80). Members of the CCGCoP practice in 48 of 50 US states in more than 250 practice settings. Results from commercial multigene panel tests on cases presented by CCGCoP members during a weekly multidisciplinary, Continuing Medical Education accredited, web-based case conference series between January 1, 2014 and September 30, 2014 by the CCGCoP are summarized in Figure 2. The seven cases detailed in the following vignettes were chosen to exemplify and discuss the challenges of multigene panel testing. All probands were ascertained through an IRB approved protocol. Cases were adjusted to anonymize the pedigrees.

## Results

The results from 348 commercial multigene panel tests ordered from January 1, 2014, through October 1, 2014, are shown in Figure 2.

### Challenging multigene panel genetic counseling risk assessment cases

#### Colorectal Cancer in *CDH1*

**Figure 3:**
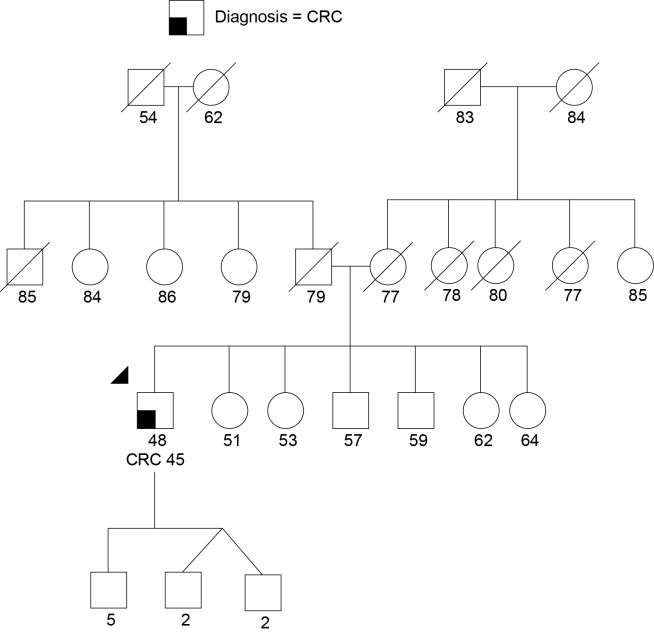
**Proband (arrow head) with colorectal cancer (CRC) and a CDH1 monoallelic mutation**. Note the large extended family without cancer. There was no known gastric cancer even in the extended family. Please see associated vignette for more details.

Case 1 is a 48-year-old male of Chinese ancestry diagnosed with metastatic left-sided adenocarcinoma of the colon at age 45. Given his young age at onset he was referred for GCRA. His family history was devoid of other cancers (Figure 3). In the absence of polyposis, his early age at diagnosis prompted the pathology laboratory to complete microsatellite instability testing and immunohistochemistry for the mismatch repair (MMR) proteins (MLH1, MSH2, MSH6, PMS2) associated with Lynch syndrome (LS), both of which returned showing no evidence of defective MMR. Given his young age, residual small risk for LS, and remaining concern for potential *MUTYH*-associated polyposis or attenuated familial adenomatous polyposis (AFAP), a multigene cancer panel was chosen to try and better understand potential hereditable cancer risk. He was found to carry a pathogenic mutation in *CDH1*, designated c.283C > T.

*CDH1* is a tumor suppressor gene that encodes epithelial cadherin. Germline mutations have been associated with hereditary diffuse gastric cancer (HDGC), a rare autosomal dominant condition historically thought to be highly penetrant, with evidence to suggest a cumulative diffuse gastric cancer risk of 80% by 80 years. Women with HGDC also have a 39–52% risk for lobular breast cancer (57, 74). Given the lack of efficacy in available screening for diffuse gastric cancer, current consensus guidelines indicate that prophylactic gastrectomy should be considered for mutation carriers (81). There is currently insufficient evidence to suggest that colorectal cancer (CRC) is part of the spectrum of HDGC-related cancers (57, 58), so the genetic finding does not appear to explain the patient’s phenotype. This specific mutation has been reported once previously and was associated with multiple individuals with invasive lobular breast cancer in a family without DGC or CRC (82).

The identification of a *CDH1* mutation in this case was considered an incidental, yet potentially meaningful test result for the family. Given the absence of stomach cancer in the family, his mutation was likely either *de novo* (a new germline mutation), or the familial penetrance of gastric cancer is low with this mutation; similar to the above family previously reported in the literature (82). Given his current poor prognosis no further management recommendations were made for him at this time. Unfortunately, both of his parents were deceased and therefore it may not be possible to further clarify whether this was an inherited mutation with low penetrance, or a *de novo* mutation. If a sibling pursues testing and tests positive, parental gonadal mosaicism (a proportion of a parent’s sperm or eggs had the mutation) could be another possibility. Either way, in this situation testing for all first-degree relatives was recommended to clarify if there are other individuals at elevated cancer risk. His children were recommended to have testing between the ages of 18 and 21. For individuals identified with the mutation, gastrectomy would need to be considered since DGC surveillance has unproven value (57). Enhanced breast screening, including annual breast magnetic resonance imaging (MRI) for female carriers, would also be recommended (75).

#### Mosaic *TP53*

Case 2 is a 42-year-old unaffected woman of Northern European ancestry referred for interpretation of ambiguous results from a breast focused multigene panel ordered by her referring physician due to her maternal family history of cancer (Figure 3); her mother had breast cancer at age 74, her aunt had colon cancer at age 76, her grandmother had leukemia at age 60, and her great-grandmother had breast cancer at age 75. There was no other family history of cancer. Results revealed a “likely pathogenic” variant in *TP53*, designated c.542G > A. However, the allele ratio deviated from 50%, suggesting the possibility of somatic mosaicism.

Li–Fraumeni syndrome (LFS) is a hereditary cancer syndrome associated with heterozygous germline mutations in *TP53* (83–85). Lifetime cancer risk is approximately 70% for male carriers and approaches 100% for female carriers (86). Although LFS includes predisposition to multiple and various primary neoplasms, the core cancers with highest risk include sarcoma, brain, breast, and adrenocortical carcinoma, there are also reported associations with colon, gastric, melanoma, bronchoalveolar, and hematological malignancies (86, 87). The only prospective observational screening study to date followed 33 LFS patients for a mean duration of 2 years (88); 18/33 underwent surveillance with their comprehensive protocol, and 10 asymptomatic tumors were found in seven individuals. Remarkably, all of the individuals in the surveillance group were living at the completion of the study (100% survival), compared to the standard (symptomatic) care group, wherein 12 high-grade/stage tumors developed in 10 LFS patients and only 2 individuals survived (20% survival) (88). For adults, the screening protocol entailed: annual whole body, breast (females only along with mammogram), and depending on the family history, dedicated brain MRI; colonoscopy every 2 years beginning at age 40; annual dermatologic exam; and a complete blood count (CBC), erythrocyte sedimentation rate, and lactate dehydrogenase for hematological malignancy screening (88). This study demonstrated the feasibility of screening these high risk patients, and this protocol has now been adopted (and adapted) nationally and internationally (75, 86). Previously, most LFS cases were identified using clinical criteria (Classic LFS, Chompret, Birch, and Eeles) (89). However, next-generation sequencing technology enabled *TP53* testing to be included in most hereditary cancer gene panels.

Given that the patient’s personal and family history did not meet criteria for a specific hereditary cancer predisposing syndrome other possibilities needed to be considered. Further discussion with the laboratory revealed that the mutation was detected in only 13% of DNA isolated from peripheral blood lymphocytes, suggesting a mosaic *de novo* finding, or hematological or other malignancy. No additional information from the laboratory or literature regarding the specific variant was available. Concern about apparent clonal hematopoiesis with a *TP53* mutation as a manifestation of an occult hematological malignancy led to recommendations for a baseline CBC (normal), as well as annual screening CBC. There were no available living family members to help clarify the results any further. Given that constitutional mosaicism for the *TP53* mutation could not be excluded, it was decided that high risk breast screening with addition of breast MRI and clinical breast exams every 6 months was justified. This approach was also supported by the fact that empiric risk model estimates indicated that the patient had moderately elevated breast cancer risk (>20%) based on her family history (90). Although often discussed in the literature, there is insufficient evidence regarding risk associated with exposure to ionizing (imaging or therapeutic) in the context of LFS (91). A colonoscopy was recommended based on the genetic finding as well as on her family history of CRC. It was discussed with the patient that her cancer risks are currently unclear given the mosaic nature of her genetic finding. She expressed distress over the test results given the uncertainty of her cancer risks and potential that the fraction of cells with the *TP53* mutation represented an incipient hematological malignancy.

#### *PMS2*, Incidental Finding but Possibly Significant for Management

Case 3 is a 30-year-old unaffected woman of Northern European ancestry, self-referred for GCRA due to a family history of multiple cancers that included a paternal aunt who had breast cancer at age 44 and a new primary breast cancer at age 46, who passed away at age 52, a maternal grandmother who had breast cancer in her 60s and died at age 70, and a maternal aunt with ovarian cancer in late 40s who was alive at age 55 (no pathological reports available). Meeting the National Comprehensive Cancer Network (NCCN) hereditary breast and ovarian cancer syndrome (HBOC) genetic testing criteria (75), the patient chose to proceed with a multigene pan-cancer panel after counseling and informed consent. This was intended to provide coverage for HBOC as well as LS given the potential association with ovarian cancer (reported in her maternal aunt). Testing revealed a complete deletion of exon 14 in *PMS2*.

*PMS2* mutations are associated with LS, a condition that increases the risk for developing CRC, uterine, ovarian, hepatobiliary, urinary tract, brain, skin, and other gastrointestinal malignancies (49, 92–95). The exact cancer risks conferred by *PMS2* mutations are unclear, but they are thought to be lower than other MMR gene mutations (96). Available evidence to date indicates that mutations in the *PMS2* gene confer a lifetime CRC risk of 15–20% (compared to lifetime risk as high as 80% with the other LS-associated MMR genes) (52). In addition, it is estimated that *PMS2* carriers have a 15% lifetime risk of endometrial cancer (compared to up to 60% with other LS-associated MMR genes). Limited data exist regarding the exact risk estimates of other extra-colonic cancers (52, 76, 96, 97).

There may be a very modest risk of breast cancer (10 years risk 2% [95% CI = 1–4%]; SIR = 1.76 [95% CI = 1.07–2.59]) following CRC among women with MMR gene mutations; however, the majority of the MMR genes in the report were MSH2 and MLH1 (both thought to be associated with greater cancer risk) (98). Therefore, the patient was counseled that the results were not explanatory of the multiple breast cancers seen in her family. Additionally, she was also counseled that the history of ovarian cancer was more likely to be unrelated to the *PMS2* mutation if there was papillary serous histology vs. endometrioid. Nonetheless, she was counseled that the detected mutation confers modestly elevated risk for CRC for her, and that testing other family members was recommended. Recommendations were made for a colonoscopy with repeat every 1–2 years per NCCN guidelines (76). She was given enhanced breast cancer screening recommendations inclusive of annual breast MRI due to her elevated lifetime breast cancer empiric risk estimate of over 20% (90, 99). Consideration of a hysterectomy with bilateral salpingo-oophorectomy was discussed but decision-making was deferred by the patient at the time of the visit. She felt upset regarding the unanticipated risk for CRC and uncertainty regarding her ovarian cancer risk.

#### Management of *PALB2*-Related Cancer Risks

**Figure 4:**
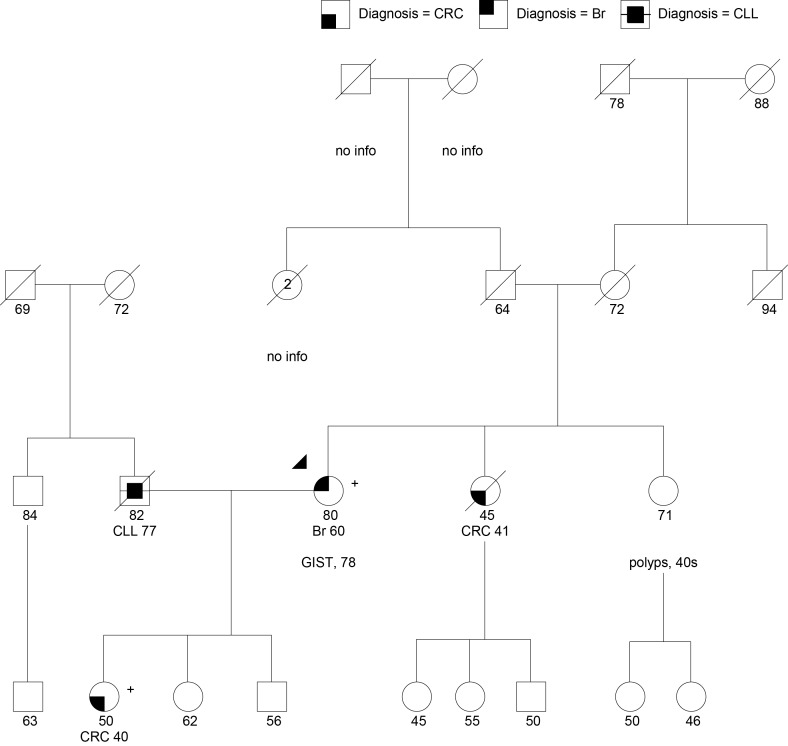
**Family with a pathogenic PALB2:c.3113G > A (p.Trp1038Ter) mutation (+) in the proband (arrow head) and daughter**. CLL, chronic lymphocytic leukemia; Br, breast cancer. *PALB2* mutations have not been associated with CRC, GIST, or colon polyps (polyps, number unknown). Please see associated vignette for more details.

Case 4 is an 80-year-old woman of Northern European ancestry referred for GCRA after her daughter was found to carry a pathogenic *PALB2* gene mutation (Figure 4). The patient had a history of breast cancer at age 60 and a gastrointestinal stromal tumor (GIST) at age 78. Her family history includes a sister with CRC at age 41, and a sister with multiple precancerous polyps since her 40s, number unknown. The patient’s daughter was diagnosed with CRC at age 40. Presumably due to her personal and family history of multiple cancers suggesting different heritable etiologies, she pursued a multigene panel at an outside hospital, which revealed a pathogenic mutation in *PALB2*, designated c.3113G > A (p.Trp1038Ter).

PALB2 (partner and localizer of BRCA2) interacts with BRCA2 in the repair of DNA double strand breaks. Biallelic mutations in *PALB2* cause Fanconi Anemia type N, characterized by growth retardation, developmental disabilities and a high risk for pediatric solid tumors (100). Monoallelic (heterozygous) mutations in *PALB2* cause an increased risk for breast cancer, with the highest risks for cases with a family history of breast cancer (31, 32, 101). The largest study of *PALB2* mutation carriers to date indicated that the cumulative risk of breast cancer at age 70 was 35% regardless of family history, whereas those with two first-degree relatives diagnosed with breast cancer before age 50 had an absolute risk of 58% by age 70 (32). *PALB2* founder mutations exist in Polish, Danish, and Russian HBOC cohorts (102–104). Antoniou et al. (32) observed an increased ovarian cancer risk for carriers by a factor of 2.3; however, findings did not reach statistical significance (32). Thus, although it is likely that there is a moderately elevated risk for ovarian cancer associated with *PALB2* mutations, the magnitude has not yet been established. *PALB2* mutations have also been identified in a small proportion of hereditary pancreatic cancer families (105, 106). The magnitude of pancreatic cancer risk conferred by *PALB2* mutations also remains unclear. Given the related pathway, it may be near the level observed in *BRCA2* mutation carriers (RR = 5.9) (107, 108), which correlates with an absolute risk of <5% lifetime. *PALB2* mutations have not been associated at this time with an increased risk for CRC or GIST.

The patient was the only one in her family with a breast cancer diagnosis. At this point, there is insufficient evidence about new primary breast cancer risk associated with *PALB2* mutations to recommend consideration of risk-reducing bilateral mastectomy, especially in a post-menopausal patient. Furthermore, there is no consensus at this point regarding consideration of risk reduction salpingo-oophorectomy or application of pancreatic surveillance in *PALB2* carriers. She was recommended to continue enhanced breast cancer surveillance. Given the lack of other cancers in the family, her ovarian cancer risk was estimated at 5–10%, and her pancreatic cancer risk was estimated to be <5%. Given the strong history of CRC and polyps in the family, the patient’s sisters were recommended to pursue their own GCRAs on the assumption that there may be a separate genetic issue for them. Despite uninformative (negative) genetic testing for the LS genes (*MSH2*, *MLH1*, *MSH6*, *PMS2*) in the patient’s daughter, she was recommended to pursue MSI and IHC for the respective MMR proteins on her CRC to help delineate the tumor phenotype, as a small proportion of such cases can show a defective MMR profile due to acquired (somatic) MMR mutations, or rarely, suggest a germline gene mutation undetectable by standard testing techniques.

#### *CHEK2*, Finding Clinical Utility

**Figure 5:**
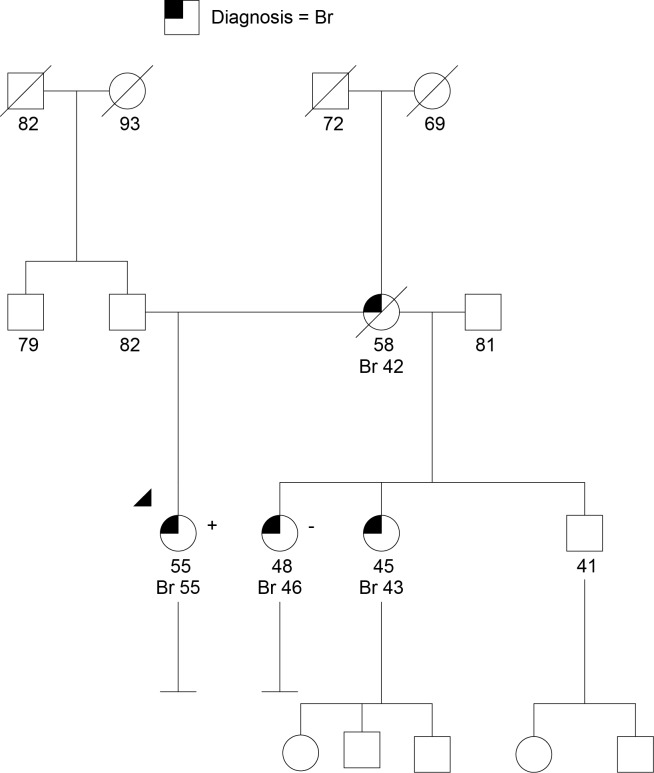
**Family with a suspected deleterious *CHEK2* allele that does not track as expected with the family history of cancer**. Breast cancers (Br) with ages are shown. The *CHEK2* mutation (+) in the denoted proband (arrow head) did not track as expected with one of the maternal half-sisters (−). Testing for other family members was not available. Please see associated vignette for more details.

Case 5 is a 55-year-old woman of Northern European ancestry with a recent history of an estrogen receptor positive (ER+) invasive lobular breast cancer and atypical ductal hyperplasia in the contralateral breast. She underwent bilateral mastectomy and was prescribed Tamoxifen. Her family history (Figure 5) was significant for a mother diagnosed with breast cancer at age 42 who succumbed to metastatic disease at age 58, and two maternal half-sisters with breast cancer (one had invasive lobular breast cancer diagnosed at age 46, the second had invasive ductal carcinoma at age 43). With ≥3 breast cancers in the family the patient met NCCN criteria for genetic testing (75). Given that both half-sisters previously had uninformative (negative) *BRCA1* and *BRCA2* testing, a multigene panel that included other breast cancer predisposition genes was offered and completed. Testing revealed a suspected deleterious splice site mutation in *CHEK2*, designated c.846 + 1G > A.

*CHEK2* encodes the checkpoint kinase 2 protein, and germline *CHEK2* mutations have been associated moderately elevated risk for breast cancer, with an odds ratio of 2.7 for unselected breast cancer cases (Table 1) (109). Evidence also suggests an approximately twofold elevated risk for CRC in *CHEK2* mutation carriers (54, 55). However, most published research to date pertains to the *CHEK2* c.1100delC founder mutation. The *CHEK2*-associated breast cancer risk of 20–30% lifetime is not generally high enough to warrant consideration of risk-reducing bilateral mastectomy, but heightened surveillance with additional annual breast MRI is recommended (75). Moreover, empiric risk estimates based on family history of breast cancer may mirror the magnitude of *CHEK2*-associated risk in breast cancer families found to have a mutation. In this case, family members already had a lifetime breast cancer risk of approximately 25% based on the Tyrer–Cuzick empiric risk model (99). Therefore, the clinical utility of testing for the known family *CHEK2* mutation in families like this is not clear.

Although it is unclear whether identifying a mutation in a moderate risk gene like *CHEK2* will change the care for family members who have elevated empiric risks already, it may illuminate cancer risks that are not apparent based on the family history alone. For example, the twofold elevated risk for CRC associated with a *CHEK2* mutation prompted a recommendation for enhanced surveillance with colonoscopy every 5 years beginning at age 40, in addition to the recommendation for annual breast MRI (75, 110). GCRA was offered for the patient’s half-siblings. Of note, one maternal half sibling with breast cancer tested negative for the mutation, illustrating the interpretive challenge associated with moderate risk genes, as they often do not track as expected within the family (Figure 5).

#### What is *NBN* Again?

Case 6 is a 67-year-old female of mixed European and Hispanic ancestry with a history of a locally advanced ER+ right breast cancer diagnosed at age 42. Family history was significant for both of her grandmothers being diagnosed with post-menopausal breast cancer in their 60s. She received a right mastectomy with adjuvant chemotherapy for treatment of her cancer and was 25 years post treatment without evidence of disease at the time of GCRA. She was self-referred to better understand the potential heritable risk for her 34-year-old daughter. NCCN HBOC genetic testing criteria was met (75). Testing was recommended and the patient chose a multigene pan-cancer panel. Testing revealed an *NBN* VUS, designated c.643C > T.

*NBN* is a low cancer risk category gene (Table 1). It encodes the Nibrin protein, which is involved in DNA damage response pathway. Historically, biallelic germline mutations in *NBN* have been associated with Nijmegen Breakage syndrome (NBS), a rare autosomal recessive disorder associated with immunodeficiency, dysmorphic features, and high risk of lymphoid malignancy (111). There is some evidence of increased cancer risks for individuals who are heterozygous (monoallelic) for the common Eastern European founder mutation c.657del5, and for those who carry the c.643C > T variant (42, 111). However, definitive evidence regarding *NBN*-associated cancer risks is lacking, and there are inconsistencies in variant classification between diagnostic laboratories. Known *NBN* mutation carriers of childbearing age should be offered prenatal counseling, given the risk for NBS if both partners of a pregnancy are found to be *NBN* carriers.

At the time of the patient’s appointment, one major lab was categorizing c.643C > T as pathogenic, and another as a likely benign variant. This highlights the need for consistency and transparency of the variant classification protocols used among different laboratories. For instance, variants of unknown significance are considered uninformative for management purposes, and should not illicit gene-specific treatment, surveillance recommendations, or testing of other family members for the same purpose (112). We explained to the patient that even if the variant is reclassified as a pathogenic mutation by the reference laboratory, current cancer screening and management recommendations would still be based on the personal and family history of cancer using empirical risk modeling. Individualized GCRA and prenatal counseling was recommended for her daughter. Although the patient expressed her appreciation for our interpretation and recommendations, she was unnerved by her ambiguous result.

#### *RAD51C* is Associated with Ovarian Cancer and …

Case 7 is a 32-year-old of Korean ancestry recently diagnosed with a multi-focal ER+ infiltrating ductal carcinoma of the right breast. Her family history includes a sister diagnosed with acute myeloid leukemia at age 27 and a maternal uncle with thyroid cancer in his 50s. She underwent a unilateral mastectomy with immediate reconstruction and was prescribed a 5-year course of Tamoxifen. She is newly married and was contemplating childbearing just before her diagnosis. As her breast cancer diagnosis was under the age of 36, she met testing criteria for both LFS and HBOC (75, 89). After genetic counseling and informed consent, the patient chose to proceed with a breast cancer-specific multigene panel. The only finding among 17 genes was a deletion involving 704 bp in the 3′ untranslated region downstream of the stop codon in *RAD51C*. The laboratory reported that the variant was of “indeterminate significance” and yet that it may increase cancer risk.

*RAD51C*, also known as Fanconi anemia complementation group O (FANCO), is part of the *RAD51* gene family and is essential for homologous recombination repair. Biallelic mutations in this gene can cause a Fanconi anemia-like phenotype, and current evidence suggests that monoallelic mutations confer a moderately increased risk for ovarian cancer (Table 1) (67, 113). However, the magnitude of breast cancer risk, if any, associated with monoallelic germline *RAD51C* mutations is uncertain (38, 113).

The commercial laboratory label of “indeterminate significance” for this variant is problematic as it does not fall into one of the five categories recommended for variant classification by the American College of Medical Genetics and Genomics and the Association for Molecular Pathology (pathogenic, likely pathogenic, benign, likely benign, or uncertain significance) (112). However, although most commercial laboratories use this or a similar variant classification scheme, variability remains, with some labs using such variant classifications as “inconclusive,” “unknown,” and “indeterminate significance.”

Regardless, VUS should be treated and reported to the patient as an uninformative finding until more information is known. However, this particular mutation was reported as a variant of intermediate significance rather than a VUS. The variant lies in the non-translated region of the *RAD51C* mRNA, therefore, it could potentially interfere with RNA processing, hence protein production/function; or it may do nothing at all to the protein. The patient was counseled about the uninformative result and ambiguous interpretation. Given the absence of ovarian cancer in the family, the ambiguous test result, and her young age, we did not recommend bilateral salpingo-oophorectomy. Additionally, we did not recommend further testing for this variant within her family at this time. A plan was made to see her again in 2–3 years, with the hope that more information will be known about this particular finding and the absolute cancer risks associated with *RAD51C*. The patient remained understandably concerned about her personal and family’s risk for future cancers, especially ovarian.

## Discussion

The above cases highlight the complexities inherent in the use of multigene panels that include low and moderate cancer risk genes, as well as the potential that a higher risk variant discordant with the personal or family cancer phenotype may be detected in some patients. GCRA, counseling, and management recommendations are often complicated by a lack of family information and mutation tracking information, a crucial component for pedigree-based studies. When pathogenic mutations in high risk genes are incidental (i.e., not associated with the presenting phenotype) (see Table 1 and *CDH1*, *TP53*, *PMS2* cases above) the results can be particularly challenging. Findings that are “off-phenotype” (not known to be associated with the particular underlying cancer) may suggest rare or novel genotype–phenotype correlations, mosaic or *de novo* findings, limitations in family structure or simply incidental findings from broad panel testing. However, one is usually compelled to make management recommendations based on genotype when pathogenic mutations in high cancer risk genes are discovered, instead of on the family history or phenotype alone. However, as in the mosaic *TP53* case above, the finding of a pathogenic variant in a highly penetrant hereditary cancer syndrome gene in only a subset of the patient’s DNA added another level of management complexity. In that particular case, after a thorough discussion with the patient regarding her various options, management was based mainly on familial empiric risks, instead of genotype alone. The heterozygous *PMS2* case demonstrates the importance of providing thorough pre-test counseling and informed consent for unanticipated results, especially when pan-cancer multigene panel testing is pursued. This case also demonstrates the obstacle of redirecting the patient’s focus on cancer risks that may not have been expected prior to testing.

Even though there may have been a selection bias toward mutation positive and VUS case accrual since some CCGCoP members selectively present higher complexity cases during case conferences, as evident in Figure 2, if historically high risk genes only were included in the panels [see Figure 2 (table), *BRCA1*, *BRCA2*, *MSH6*, *PMS2*, *TP53*, *APC*, *CDH1*], the results would have been positive only 6.2% of the time, instead of 17%. Furthermore, results returned with VUS 42% of the time, likely due in large part to the testing inclusion of more genes and the current knowledge gap in human genetic variation. Taken as a whole, these figures stress the importance of adequate pre-test counseling in anticipation of higher percentages of positive, VUS, unexpected, and ambiguous test results.

Will it change medical and/or surgical management? Can screening and/or surveillance be altered? Currently, consensus guidelines to answer these questions are lacking for many moderate and low risk cancer predisposing genes included in many commercially available multigene panels. Figure 1 can be used as a framework to help categorize high, moderate, and low risk cancer predisposing genes. High risk gene mutations are thought to explain specific cancer phenotypes; however, moderate and low risk genes are likely not the sole explanation for the cancer in the individual, and/or family. Management of pathogenic mutations in moderate risk genes is difficult and requires an evaluation of the personal and family history of cancer (see Table 1 and *PALB2* and *CHEK2* cases above). Currently, it is not clear how to use low cancer risk genes in management and risk counseling (i.e., see Table 1; *NBN*, *XRCC2*, *GALNT12*, etc.), since recommendations should be based either way on the personal and family history of cancer.

As our collective knowledge base expands, we will also learn how specific mutations or other genetic modifiers, such as single nucleotide polymorphisms or epigenetic factors, alter risk. As noted in the *CHEK2* and *NBN* cases above, outside of specific founder mutations, cancer-related risks for these genes remain ambiguous. Even though *ATM* is considered a moderate risk (20–25%) breast cancer predisposition gene, two specific mutations (c.7271T > G and IVS10-6T > G) were estimated to confer a 60% cumulative lifetime risk of breast cancer (114, 115). Similarly, although *BRIP1* has been associated with only a modestly increased risk for ovarian cancer, an Icelandic mutation (c.2040_2041insTT) has been associated with an eightfold risk for ovarian cancer (116). The significant knowledge gaps in genotype–phenotype correlations, expressivity, and penetrance will only be unraveled by marrying thorough and relevant clinical data with genetic findings. This emphasizes the need for community-based clinicians to contribute genotype/phenotype data generated by multigene panels to large national and international hereditary cancer collaborative research registries. Some current initiatives include the evidence-based network for the interpretation of germline mutant alleles (ENIGMA), the Prospective Registry of MultiPlex Testing (PROMPT), and the consortium of investigators of modifiers of BRCA1/2 (CIMBA) among others (117–119). Laboratories must also openly contribute their findings to public databases, such as ClinVar (120).

In summary, multigene hereditary cancer panel testing can lead to unexpected and complex findings. This stresses the importance of appropriate pre-test counseling and informed consent by a knowledgeable genetics professional (19). Additionally, choosing a phenotype-specific panel with high clinical utility/risk genes instead of pan-cancer panels inclusive of many “off-phenotype” and low risk genes can decrease the amount of incidental and uncertain results. If ambiguity is found on testing, many resources, including the literature, reference laboratory, colleagues, and national experts, are available to help better clarify counseling and management for the patient and family. In the case of uninformative testing, or in the case of mutations in low and moderate risk genes, empiric risk modeling may help guide management. Appropriate prenatal counseling and partner testing is advised in situations involving mutations in genes that carry a recessive disease risk (those in the Fanconi anemia pathway, *NBN*, *ATM*, etc.). In challenging cases, patient follow-up every 1–3 years may be prudent until the patient and family recommendations can be better clarified. Multigene panels are here to stay, therefore, we must collectively continue to clarify the absolute and relative cancer risks, delineate genotype–phenotype correlations, and reclassify variants of unknown significance. This can only be done with laboratory transparency of testing results and through the help of collaborative research studies that merge genetic findings with phenotypic data.

## Conflict of Interest Statement

The authors declare that the research was conducted in the absence of any commercial or financial relationships that could be construed as a potential conflict of interest.

## Funding

Research reported in this publication was supported by the National Cancer Institute of the National Institutes of Health under Award Numbers P30CA33572 and R25CA171998 (Pls: KRB and JNW) and RC4CA153828-01 (PI: JNW). The content is solely the responsibility of the authors and does not necessarily represent the official views of the National Institute of Health.
