# Corrigendum: Clinical Application of Multigene Panels: Challenges of Next-Generation Counseling and Cancer Risk Management

**DOI:** 10.3389/fonc.2015.00271

**Published:** 2015-12-02

**Authors:** Thomas Paul Slavin, Mariana Niell-Swiller, Ilana Solomon, Bita Nehoray, Christina Rybak, Kathleen R. Blazer, Jeffrey N. Weitzel

**Affiliations:** ^1^Division of Clinical Cancer Genetics, Department of Medical Oncology, City of Hope, Duarte, CA, USA

**Keywords:** genetics, multigene panels, genetic counseling, BRCA1

On page 4 of our manuscript, line 528, there is an erroneous reference for Figure 3.

**“Mosaic TP53**

Case 2 is a 42-year-old unaffected woman of Northern European ancestry referred for interpretation of ambiguous results from a breast focused multigene panel ordered by her referring physician due to her maternal family history of cancer (Figure 3);”

Figure 3 only pertains to the *CDH1* case above (correctly referenced there). Therefore, the sentence should read:

“Case 2 is a 42-year-old unaffected woman of Northern European ancestry referred for interpretation of ambiguous results from a breast focused multigene panel ordered by her referring physician due to her maternal family history of cancer;”

We apologize for any confusion this may have caused.

## Conflict of Interest Statement

The author declares that the research was conducted in the absence of any commercial or financial relationships that could be construed as a potential conflict of interest.

